# Factor structure and gender invariance of the Beck Depression Inventory – second edition (BDI-II) in a community-dwelling sample of adults

**DOI:** 10.1080/21642850.2020.1715222

**Published:** 2020-01-20

**Authors:** André Faro, Cicero R. Pereira

**Affiliations:** a Department of Psychology, Federal University of Sergipe, UFS, São Cristóvão, Sergipe, Brazil; b Department of Psychology, Federal University of Paraiba, UFPB, João Pessoa, Paraíba, Brazil

**Keywords:** Beck depression inventory – Second edition (BDI-II), confirmatory factor analysis (CFA), measurement invariance (MI), depressive disorders, bifactor analysis

## Abstract

**Objective**: The main purpose of this study was to investigate the factor structure of the Beck Depression Inventory – Second Edition (BDI-II) in a sample of adults. Specifically, we evaluated the BDI-II based on confirmatory factor analysis of different measurement models, and compared the optimal factor structure of the BDI-II by gender using measurement invariance analysis.

**Method**: A cross-sectional survey with 717 community-dwelling adults was conducted. The Brazilian Portuguese version of the BDI-II was administered. Seven different models (one-, two-, three-factor models and their bifactor structures) were tested through CFA. CFA and multigroup analysis were executed with the software MPLUS (Weighted Least Squares Estimator – WLSMV).

**Results**: Four bifactor models reached acceptable fit indices. A bifactor model with two specific factors (Cognitive–Affective, and Somatic-Affective) provided the best fit to the data. The multigroup analysis of this model demonstrated invariance by gender.

**Conclusions**: Our findings support the use of the total BDI-II score to identify depressive symptoms, including gender comparisons. Since a bifactor structure fit the data better, the scores of the specific factors should not be used as the first choice, or at least should be used with caution. The analysis of the severity of depression, based on a total score, seems to be the most appropriate option.

## Introduction

The World Health Organization (WHO) estimates that more than 300 million people live with depression in the world (World Health Organization (WHO), 2017). If not properly diagnosed and treated, depression tends to aggravate people's suffering over time and increase the number of years lived with disability and years of life lost (Siu et al., 2016). Depression is the second-leading cause of years lived with disability worldwide (Hay et al., 2017), with prevalence close to 5% per year (World Health Organization (WHO), 2017). Because of its remarkable impact on health, valid and reliable psychometric instruments are needed, especially to improve population screening for early detection and proper referral of suspected cases of depression (Ferrari et al., 2013).

The Beck Depression Inventory – Second Edition (BDI-II) is one of the most widely used instruments for screening the presence and severity of depressive disorder symptoms in clinical and nonclinical settings (Huang & Chen, 2014; Wang & Gorenstein, 2013a). The BDI-II is consistent with the diagnostic criteria of the Diagnostic and Statistical Manual of Mental Disorders – 4th edition (DSM-IV) and can be applied to subjects from 13 years old (Beck, Steer, & Brown, 1996; Gorenstein, Pang, Argimon, & Werlang, 2011). The BDI-II comprises 21 items answered on a 4-point scale about the occurrence of depressive symptomatology in the previous two weeks. It is an instrument suitable for use in community screening and in studies with large samples, given that this scale is relatively short, self-administered, and has an easy scoring procedure. Results indicate that the BDI-II shows high reliability and internal consistency in several populations (Wang & Gorenstein, 2013a).

The BDI-II has been translated from English into many languages (e.g. Arabic, Chinese, Korean, Turkish, Croatian, Japanese, Xhosa, Malay, Spanish, Norwegian, Icelandic, Persian, Swedish, Welsh, German, Lithuanian, French, Dutch, and Portuguese; see Huang & Chen, 2014; Wang & Gorenstein, 2013a), which has contributed to confirming its applicability in a large range countries, cultures, and sample profiles. However, in spite of being widely accepted as a good parameter for evaluating depression symptomatology, findings about the factor structure of the BDI-II are not conclusive (McElroy, Casey, Adamson, Filippopoulos, & Shevlin, 2018). This is a usual question in many studies because after the BDI release in 1961, Beck, Steer, and Brown (Beck et al., 1996) mentioned that the items’ arrangement may vary according to the characteristics of the sample or psychometric parameters of the factor extraction.

The BDI-II items assess somatic, affective, and cognitive domains of the construct of depression. From the items’ array, it is common to find two- and three-factor oblique models in the BDI-II literature, both in clinical and nonclinical samples. For example, the best-known models were proposed by Beck et al., (1996)., where they identified a (1) Somatic-Affective and (2) Cognitive factors in a clinical outpatient sample, and a (1) Cognitive–Affective and (2) Somatic factors in a college student sample. Also commonly replicated in further investigations, we found three other models with a two-factor structure. Dozois, Dobson, and Ahnberg, (1998) found that (1) Cognitive–Affective and (2) Somatic-Vegetative factors emerged from an undergraduate student sample, and Steer, Ball, Ranieri, and Beck, (1999) found (1) Cognitive and (2) Non-Cognitive factors in a sample of depressed adult outpatients. Of the family of three-factor models, some have received particular attention. Osman, Downs, Barrios, Kopper, Gutierrez, and Chiros (Osman et al., 1997) and Wu (2010) detected (1) Negative Attitudes, (2) Performance Difficulty and (3) Somatic Elements as factors in samples of undergraduate and high school students, respectively. It is noteworthy that although both these studies equally label the models, the item allocation per factor does not exactly match. For instance, the Somatic Elements factor contains four items in Osman et al. (1997) and five items in Wu (2010). Furthermore, Buckley, Parker, and Heggie, (2001), and Beck, Steer, Brown, and Does, (2002) found (1) Cognitive, (2) Affective, and (3) Somatic factors, but the items’ arrangement was not the same again.

More recently, bifactor measurement models have become a popular alternative for testing the structure of the BDI-II. Bifactor analysis tests the hypothesis of a general factor (G-factor) that explains most of the common variance extracted simultaneously from the items under an orthogonal design and its specific factors (García-Batista, Guerra-Peña, Cano-Vindel, Herrera-Martínez, & Medrano, 2018; Heinrich, Zagorscak, Eid, & Knaevelsrud, 2018). Most investigations perform bifactor analysis from two- and three-factor BDI-II models, which means the researchers only added a general factor to the *n*-factor model. For example, Brouwer, Meijer, and Zevalkink, (2013) showed that a bifactor structure fitted better to the data than a set of correlated first-order specific factors. García-Batista et al. (2018) similarly found that a bifactor model is more suitable than four alternative models describing only specific factors.

Although bifactor models have performed better than specific factors models, Eid, Geiser, Koch, and Heene (2017) described several anomalous results in G-factor models when estimated with the current procedure to specify bifactor structures, which include statistical identification issues and uninterpretable estimated parameters of specific factors. As a solution, they proposed an innovative approach for estimation of bifactor models whose main characteristic is one of two procedures (named *S-1* and *S.I-1* models, respectively). The first one takes a specific factor as reference domain, to which the other domains are compared (*S-1* models). The second way is less restrictive because it takes one item as reference indicator to which the other items are compared (*S.I-1* models). Heinrich et al. (2018) estimated an *S-1* bifactor model for BDI-II and showed not only better fit to the data than several alternative models, but also more reliable estimated parameters and absence of statistical identification problems or non-convergence issues. These results are important because they indicate that estimating general factors in bifactor models needs previous definition of an item or a specific factor as a reference domain, which has not been done in previous studies.

So far, all those models have shown evidence of validity in specific contexts, for example, in particular local groups (e.g. clinical or nonclinical subjects) or in cross-cultural differences of similar groups (e.g. students in different countries). Nevertheless, such investigations are usually (or relatively) homogeneous to sociodemographic or clinical characteristics, which makes extrapolating the findings to a general population relatively limited. Moreover, although many studies replicate different structural models, reviews have not confirmed that one model is clearly better than another regarding the psychometric qualities in any population (Huang & Chen, 2014; Wang & Gorenstein, 2013a). Hence, it is still relevant to search for more appropriate models in samples of community-dwelling individuals (for example, Campos & Gonçalves, 2011), mainly because the capacity of generalization is especially important for studies in mental health and requires caution in designing studies.

To the best of our knowledge, in Brazil, there is only one study applying exploratory factor analysis (EFA) of the BDI-II among community-dwelling adults. It was published in the Brazilian BDI-II Manual (Gorenstein et al., 2011). Furthermore, as far we know, factor structure (confirmatory factor analysis [CFA]) or even scrutiny of concurrent models of the BDI-II in Brazil has not been sufficiently analyzed yet. For instance, Gorenstein et al. (2011) performed an EFA with a full sample composed of many different groups, among them 182 community-dwelling adults. They found a two-factor model ([1] Cognitive–Affective and [2] Somatic-Affective), but item 10 did not load in any factor within the referred adult sample. Also, we did not find any study replicating the two-factor model developed by Gorenstein et al., either EFA, or CFA.

Exclusively from scientific journals, we only found two studies on the validation of the BDI-II in Brazil. First, Paranhos, Argimon, and Werlang, (2010) analyzed the psychometric properties of the BDI-II in a sample of adolescents. They found a model consisting of two-factors ([1] Cognitive and [2] Somatic-Affective), with the same labeled factors of Beck et al., (1996), but with a distinct item arrangement. Finally, Finger and Argimon (2013) detected a three-factor model in undergraduate students ([1] Cognitive-Emotional, [2] Somatic, and [3] Loss), which was distinct from other models in the literature. Based on this scenario, investigations of the BDI-II structure in Brazil are still relevant because there is no sufficient evidence of factor validity using community-dwelling adult samples. Indeed, there is no CFA of a Brazilian BDI-II model with any sample.

Another important aspect understudied with BDI-II is the suitability of its factor structure to measure adequately depression across gender. This is a relevant aspect because differences between males and females are considered to be an evident health disparity. For instance, a recent meta-analysis with a representative sample of almost two million men and women worldwide found that: (1) females have higher rates of depressive symptoms and major depression than males; (2) the gender discrepancies are relatively stable throughout life span; and (3) the differences of the magnitude of depression by gender are not significantly explained by economic development or nation-level wealth (Salk, Hyde, & Abramson, 2017). These findings reinforce the specific impact of the gender variable for understanding depression’s occurrence (World Health Organization (WHO), 2017; Salk et al., 2017; Parker & Brotchie, 2010).

Despite the centrality of gender disparities in depression, research has usually focused gender differences based on the comparison of scores of depressive symptoms, paying less attention to the equivalence of measures between gender groups. For establishing the reliability of group difference in depression, the measurement instrument must be able to measure the presence of symptoms in an equivalent way between gender groups. That is, it is necessary to assume that the instrument measures the same attributes (mean, scores, factors, etc.) in the same way for both males and females (Milfont & Fisher, 2010). Therefore, measurement invariance (MI) is an important procedure to test the adequacy of scales when CFA is performed, and it has been recommended in multigroup analysis of the BDI-II (Dere et al., 2015; Moore, Neale, Silberg, & Verhulst, 2016; Wu, 2017; Wu & Huang, 2014). For group comparisons, MI is used to verify whether an instrument has sufficient equivalent psychometric properties to state that the score differences are not due to measurement bias and properly reflect true differences in the sample. In the absence of invariant models, conclusions about differences between groups are not reliable because the models may not correspond to each other (Milfont & Fisher, 2010).

Up to this date, we found no information concerning measurement equivalence of BDI-II in a Brazilian community-based sample across gender. Previous studies in other countries have found evidence of full or partial invariance at the items level of the BDI-II between males and females (Whisman, Judd, Whiteford, & Gelhorn, 2012 Wu, 2010; Wu & Huang, 2014), but there is no consensus on which items show distinct functioning regularly. Thus, considering the importance of gender for understanding depression and due to inconclusive findings on gender invariance with the BDI-II, MI analyses in Brazil seem to be a relevant target.

In sum, the literature indicates that the findings on the factorial structure of BDI-II are still inconclusive, especially in non-clinical populations. This gains relevance in the Brazilian context, given the absence of publications with this proposal. The research that has come closest to answering this question suggests a bifactor model as having the best fit (McElroy et al., 2018; Brouwer et al., 2013; Dere et al., 2015, Azevedo R et al. 2016, Subica et al., 2014). However, as warned by Eid et al. (2017), these studies have given little or no attention to correct anomalies in the estimation of bifactor models, which raises doubts about the quality of the estimated parameters in favor of the bifactor models obtained so far.

Given the hypothesis that these limitations can be overcome, we intend to fill the following gaps: absence of BDI-II bifactor model tests according to recommendations of Eid et al. (2017) (SI-1 model) and absence of studies on confirmatory factor analysis of BDI-II in the Brazilian scenario. Additionally, by including the invariance analysis by gender, we also expect to contribute to the assessment of BDI-II equivalence between men and women. Finally, the main purpose of this study was to investigate the factor structure of the Beck Depression Inventory – Second Edition (BDI-II) in a sample of adults. Specifically, we evaluated the BDI-II based on confirmatory factor analysis of different measurement models; and compared the optimal factor structure of the BDI-II by gender using measurement invariance analysis.

## Method

### Sampling

This was a cross-sectional survey with community-dwelling adults in Aracaju (Sergipe, Brazil). Data were collected in 2017 from households in 12 neighborhoods, with only one participant per house. Initially, three neighborhoods per geographic region and two central avenues in each neighborhood were randomly selected. We controlled the sampling for gender proportion (approximately 50/50%), visited residences (two houses were skipped in each visit), and period of the day (collection in the morning, afternoon, and evening, including weekends). Subjects were invited to participate in their own homes after the presentation of the study objectives by a member of the research team. Participants were informed that they would be part of a mental health survey and would receive some psychological questions for response. They completed a printed questionnaire on their own, and the research team member intervened only when there were requests for clarification. At the end, there was a verification of unanswered questions or clarification of possible doubts. If there were no problems, the data collection procedure was terminated.

### Participants

The final sample was composed of 717 adults [confidence interval (CI) = 95%; sampling error = 3.5%], with 55.9% (*n* = 401) females and mean age of 36 years (SD = 12.79). The predominant educational level was high school (44.1%; *n* = 316), followed by graduates (42.7%; *n* = 306), and elementary school (13.2%; *n* = 95). Only 10.5% (*n *= 75) were smokers and 19.1% (*n *= 137) declared suffer from some chronic disease. Most parts were employed at the period of the data collection (79.5%; *n *= 570). The refusal rate was below 1%. Visitors, domestic workers, and subjects under 18 years or over 65 years were not eligible for our sample. The Research Ethics Committee of (omitted information) approved this study and all participants provided written consent.

### Measure

*Beck Depression Inventory – II (BDI-II).* BDI-II is composed of 21 items that comprise somatic, cognitive, and affective symptoms related to depression. The scale provides a total score and a grade on the depression severity (subclinical, mild, moderate, and severe). The BDI-II is answered on a scale ranging from 0 to 3, with a final score between 0 and 63 points. The higher the score, the greater the symptoms’ severity (Gorenstein et al., 2011).

### Data analysis

Descriptive analysis was performed using SPSS (v. 22). CFA was conducted with MPLUS (v. 6.0) using the weighted least squares estimator (WLSMV). The quality of fit was examined with the following indices: chi-square ratio (*χ*²/df), below 5 considered good fit; comparative fit index (CFI) and Tucker-Lewis index (TLI), both expected >.95 (Bentler, 1990; Tucker & Lewis, 1973); and root mean square error of approximation (RMSEA), with expected values of ≤.050 (CI = ≥.001 – ≤ .080) and *p*-close >.05 (Steiger, 1990).

By means of multiple CFA, we tested seven competing models in this study. Initially, six original multidimensional models were selected to be compared (Models II to VII), plus a unidimensional structure (Model I). We chose four two-factor and two three-factor models developed with adult samples, and commonly used in the international literature on BDI-II. Except for Model I, all models were also converted to bifactor structures. The original sample, factors, and respective item arrangement of those models are listed as follows.

*Model I.* Unidimensional: all 21 items linked to a single factor.

*Model II*. (Beck et al., 1996) (two-factor, clinical adult outpatients): Somatic-Affective (4, 10–13, 15–21) and Cognitive (1–3, 5–9, 14).

*Model III*. (Dozois et al., 1998) (two-factor, undergraduate students): Cognitive–Affective (1–3, 5–9, 13–14) and Somatic-Vegetative (4, 10–12, 15–21).

*Model IV*. (Steer et al., 1999) (two-factor, depressed adult outpatients): Cognitive (2–3, 5–9, 14) and Non-Cognitive (1, 4, 10–13, 15–21).

*Model V*. (Gorenstein et al., 2011) (two-factor, varied sample): Cognitive–Affective (1–10, 12, 14) and Somatic-Affective (11, 13, 15–21).

*Model VI*. (Finger & Argimon, 2013) (three-factor, undergraduate students): Cognitive–Affective (1–3, 5–10, 13–14), Somatic (11, 15–20), Loss (4, 12, 21).

*Model VII*. (Osman et al., 1997) (three-factor, undergraduate students): Negative Attitudes (1–3, 5–10, 14), Performance Difficulty (4, 12–13, 15, 17, 19–20), and Somatic Elements (11, 16, 18, 21).

In order to guarantee reliable estimated parameters and avoid statistical identification issues in the BDI-II general factor, we followed the recommendations Eid et al. (2017) when specifying bifactor models. Thus, we specified a less restrictive G factor model (i.e. *S.I-1* model) by previously defining the BDI-II item 20 (Tiredness or Fatigue) as an indicator of the reference domain needed to estimate the G factor. We selected this item on the base of a preliminary analysis of all items aiming to verify which one could work better as a reference item. We specifically selected this item because it has the best empirical distribution among the four response alternatives, has a high R-squared on the G-factor, presented no convergence problem, and refers to a typical somatic symptom of depression. From this procedure, we assumed that item 20 was a good indicator for the reference domain of the G-factor. The chosen reference indicator is not allowed to have loading on a specific factor because it is a measured variable that will function as a parameter for the G factor estimation. Hence, it cannot be loaded in a specific factor to ensure that the estimated model will actually be a bifactor one (see Eid et al., 2017, p. 12, for technical details).

Invariance analysis by gender of the best-fit model was performed through three levels of analysis: configural, metric, and scalar invariances. Configural invariance tested if the arrangement of items and factors was the same between groups. Metric invariance verified if the standardized regression weights were equivalent for men and women. Scalar invariance checked if the latent means scores could be properly compared (Milfont & Fisher, 2010). We used as parameters for invariance rejection Delta CFI (ΔCFI ≤ .01) and Delta RMSEA (ΔRMSEA ≤ .015) (Chen, 2007).

## Results

Table 1 shows descriptive statistics of the BDI-II items and total score. We found high Cronbach’s alpha for the total scale (*α* = .93) and the corrected item-total correlations ranged from .46 (item 10, crying) to .70 (items 4, loss of pleasure, and 12, loss of interest). For males and females, the item with the highest mean was 16 (change in sleeping habits, .82 and .85, respectively). On the other hand, item 9 (suicidal thoughts) presented the lowest mean for both males (.20) and females (.21). The items with most answers confirming the presence of the symptom were 16 (change in sleeping habits, 58.6%) and 18 (changes in appetite, 48.5%). The least mentioned items were 9 (suicidal thoughts, 13.5%) and 3 (failure, 23.0%). Such findings are similar to those of other studies with samples based on nonclinical populations (Beck et al., 1996; Beck et al., 2002; Brouwer et al., 2013; Gorenstein et al., 2011; Kojima et al., 2002; Lee, Lee, Hwang, Hong, & Kim, 2017 Osman et al., 1997;).

**Table 1.** Descriptive statistics for the Beck Depression Inventory-II items in a community-dwelling sample of adults.

		Mean (standard deviation)					
	%	Total	Male	Female	rit	Skewness	Kurtosis
1. Sadness	28.7	.38 (.69)	.36 (.72)	.39 (.67)	.66	2.10	4.42
2. Pessimism	29.1	.40 (.72)	.35 (.68)	.44 (.75)	.68	2.02	3.86
3. Failure	23.0	.37 (.75)	.38 (.76)	.37 (.75)	.64	1.99	2.97
4. Loss of pleasure	39.6	.57 (.83)	.50 (.82)	.62 (.84)	.70	1.51	1.62
5. Feeling of guilt	42.0	.51 (.69)	.48 (.72)	.54 (.68)	.60	1.45	2.26
6. Feeling of punishment	29.5	.55 (.96)	.55 (.96)	.55 (.97)	.63	1.64	1.33
7. Disconformity with oneself	19.8	.34 (.75)	.35 (.80)	.33 (.73)	.67	2.22	3.89
8. Self-criticism	47.4	.63 (.78)	.62 (.81)	.64 (.76)	.62	1.19	.99
9. Suicidal thoughts	13.5	.20 (.58)	.20 (.57)	.21 (.60)	.63	3.34	11.31
10. Crying	34.9	.64 (.98)	.54 (.99)	.71 (98)	.46	1.30	.32
11. Agitation	44.1	.69 (.93)	.65 (.93)	.72 (.94)	.56	1.27	.58
12. Loss of interest	35.7	.48 (.76)	.48 (.82)	.49 (.72)	.70	1.71	2.57
13. Indecision	45.5	.71 (.93)	.67 (.93)	.73 (.95)	.61	1.22	.49
14. Devaluation	21.5	.33 (.69)	.34 (.72)	.32 (.67)	.67	2.05	3.24
15. Loss of energy	52.0	.64 (.72)	.56 (.70)	.70 (.73)	.57	1.06	1.09
16. Changes in sleeping habits	58.6	.84 (.86)	.82 (.86)	.85 (.86)	.49	.77	−.19
17. Irritability	27.0	.51 (.78)	.47 (.80)	.54 (.76)	.63	1.66	2.35
18. Changes in appetite	48.5	.64 (.77)	.65 (.80)	.64 (.76)	.55	1.14	.87
19. Difficulties in concentration	46.0	.67 (.83)	.55 (.82)	.76 (.85)	.65	1.01	.06
20. Tiredness or fatigue	33.4	.74 (.84)	.62 (.81)	.84 (.85)	.60	1.01	.39
21. Loss of sexual interest	28.3	.42 (.78)	.28 (.65)	.55 (.86)	.50	1.90	2.84
Total score*	-	11.3 (10.95)	10.4 (11.05)	12.0 (10.84)		1.52	2.28

Notes. % = percentage of subjects who endorse options 1, 2, or 3 (presence of the symptom). r_it _= total sample corrected item-total correlation. * Cronbach´s alpha = .93.

Table 2 describes the fit statistics of the CFA. All seven models were tested and four bifactor models (II, III, V, and VI) reached acceptable fit indices. Although we found very similar statistics among those four models, the bifactor model based on the proposal of Gorenstein et al. (2011) (Model V) was slightly superior. It is composed of two primary dimensions (Cognitive–Affective [CA] and Somatic–Affective [SA]) and showed the lowest chi-square ratio in comparison to the others (*χ*²/df = 3.1), as well as better fit in the RMSEA (.054; CI .049 – .059; *p *= .099), CFI and TLI (.974 and .968, respectively).

**Table 2.** Fit Indices of the BDI-II from confirmatory factor analysis in a Brazilian adult community-dwelling sample.

Models (M)	χ² (df)	χ²/df	RMSEA (CI 95%)	*p*-close	CFI	TLI
MI Unidimensional	787.131 (189)	4.1	.066 (.062 – .071)	< .001	.956	.952
MII Two-factor	638.727 (188)	3.4	.058 (.053 – .063)	.005	.967	.963
Bifactor	536.734 (169)	3.2	.055 (.050 – .060)	.054	.973	.967
MIII Two-factor	625.323 (188)	3.3	.057 (.052 – .062)	.010	.968	.964
Bifactor	530.839 (169)	3.1	.055 (.049 – .060)	.071	.974	.967
MIV Two-factor	705.990 (188)	3.7	.062 (.057 – .067)	< .001	.962	.958
Bifactor	600.671 (169)	3.5	.060 (.055 – .065)	.001	.969	.961
MV Two-factor	658.773 (188)	3.5	.059 (.054 – .064)	.001	.966	.962
Bifactor	523.127 (169)	3.1	.054 (.049 – .059)	.099	.974	.968
Bifactor (modified)*	*428.281* (*172)*	*2*.*5*	*.046* (*.040 – .051)*	.*908*	.*981*	.*977*
MVI Three-factor	595.052 (186)	3.2	.055 (.050 – .060)	.037	.970	.966
Bifactor	529.528 (169)	3.1	.055 (.049 – .060)	.075	.974	.967
MVII Three-factor	640.314 (186)	3.4	.058 (.053 – .063)	.003	.967	.963
Bifactor **	-	-	-	-	-	-

Notes: Highlighted in Italics: Model with the best-fit indices.

* Bifactor Model V (modified): items 15, 16, 18 and 21 associated only to the General Factor. CA factor: items 1–10, 12, and 14. SA factor: items 11, 13, 17, and 19. Item 20: specified as reference item for proper estimation of the G factor in bifactor models (see Eid et al., 2017).

** Bifactor Model VII did not converge due to negative covariance matrix of three parameters.

Based on a detailed examination of the bifactor Model V, four items were detected with non-significant standardized regression weights (factor loadings) in the SA dimension, but with significant factor loadings in the General (G) factor (items 15, 16, 18, and 21). Taking into account these unnecessary parameters in the model, as well as that their removal would not change the theoretical rationale of the BDI-II, we decided to perform a new CFA of the bifactor without the referred non-significant standardized regression weights. In the new CFA, items 15, 16, 18, and 21 remained only in the G factor. The SA factor continued with four of its original items (items 11, 13, 17, and 19) and the CA had no changes in its structure (items 1–10, 12, and 14). The best-fit model was labeled bifactor (modified) and showed an important improvement in all indices (*χ*²/df = 2.5; RMSEA [.046; CI .040 – .051; *p *= .908], CFI = .981, TLI = .977). No specification errors or other modifications of the items’ structure were required in this last model.

The standardized regression weights of the bifactor Model V (modified) varied from .24 (item 10) to .65 (item 3) in the CA factor. The SA factor had variation from .28 (item 19) to .44 (item 13). In the G factor, factor loadings ranged from .50 (item 5) to .78 (item 12) (Table 3). Finally, we evaluated the gender (male or female) multigroup analysis of invariance of the bifactor Model V (modified), which showed configurational, metric, and scalar invariance across gender groups (ΔCFI ≤ .01 and ΔRMSEA ≤ .015) (Table 4).

**Table 3.** Standardized regression weights (factor loadings) of the BDI-II items.

Items	Bifactor Model V (modified) Dimensions								
	General			Cognitive-Affective			Somatic-Affective		
	T	M	F	T	M	F	T	M	F
1. Sadness	.60	.63	.58	.58	.59	.57	–	–	–
2. Pessimism	.59	.58	.62	.62	.63	.61	–	–	–
3. Failure	.56	.55	.62	.65	.69	.56	–	–	–
4. Loss of pleasure	.72	.73	.72	.36	.37	.35	–	–	–
5. Feeling of guilt	.50	.48	.54	.54	.65	.42	–	–	–
6. Feeling of punishment	.63	.62	.67	.41	.45	.32	–	–	–
7. Disconformity with oneself	.68	.71	.68	.53	.52	.49	–	–	–
8. Self-criticism	.59	.58	.64	.40	.48	.26	–	–	–
9. Suicidal thoughts	.61	.66	.60	.65	.63	.64	–	–	–
10. Crying	.53	.55	.52	.24	.23	.23	–	–	–
11. Agitation	.59	.50	.69	–	–	–	.37	.54	.16
12. Loss of interest	.78	.83	.76	.30	.27	.30	–	–	–
13. Indecision	.63	.68	.62	–	–	–	.44	.43	.42
14. Devaluation	.74	.79	.73	.40	.32	.42	–	–	–
15. Loss of energy	.76	.74	.75	–	–	–	–	–	–
16. Changes in sleeping habits	.65	.68	.74	–	–	–	–	–	–
17. Irritability	.68	.65	.71	–	–	–	.38	.43	.31
18. Changes in appetite	.71	.64	.72	–	–	–	–	–	–
19. Difficulties in concentration	.72	.70	.74	–	–	–	.28	.23	.34
20. Tiredness or fatigue	.77	.74	.77	–	–	–	–	–	–
21. Loss of sexual interest	.70	.74	.63	–	–	–	–	–	–

Notes: All items with statistically significant factor loadings (*p*-value < .05).

T = Total sample; M = Males; F = Females.

**Table 4.** Goodness-of-fit indices for invariance analysis of the BDI-II Model across gender groups.

Model	*χ*^2^	df	*χ*²/df	CFI	ΔCFI	RMSEA (CI 95%)	ΔRMSEA
Configural	603.950	340	1.8	.981	–	.047 (.040 - .053)	–
Metric	577.830	378	1.5	.986	.005	.038 (.032 - .045)	.009
Scalar	716.300	420	1.7	.979	.007	.044 (.039 - .050)	.006

Notes: df = degrees of freedom; CFI = comparative fit index; TLI = Tucker-Lewis fit index; RMSEA = root mean square error of approximation; CI = confidence interval.

## Discussion

From the household sample of community-dwelling adults, we performed several CFAs to evaluate the fit of different structured models of the BDI-II (one-, two-, and three-factor, plus their bifactor versions). Initially, four models showed good fit indices (II, III, V, and VI) and two of them (V and VI) were proposed based on Brazilian samples. Model V fitted the data and its bifactor version showed a better fit than the other models. Based on the deletion of regression parameters of four items with non-significant standardized regression weights in the SA factor, but statistically significant in the G factor, we defined the bifactor Model V (modified) as the optimal model in this study. We also analyzed the MI by gender of this model, which was non-invariant.

Conventional bifactor models have been considered to have better factor structures in CFA investigations of the BDI-II compared to unidimensional, correlated factors or high order factors (Brouwer et al., 2013; Dere et al., 2015; García-Batista et al., 2018; Heinrich et al., 2018; Subica et al., 2014). We followed an innovative procedure proposed by (Eid et al., 2017) aiming to estimate more reliable parameters for the BDI-II bifactor structure. Our results support previous bifactor findings and demonstrate that using an overall score is a proper way to analyze depressive symptomatology with the BDI-II. This score is applied to measure the presence of significant symptoms, which is represented by the general factor and usually accounts for the severity of depression (mild, moderate, and severe, for instance) (Brouwer et al., 2013; Subica et al., 2014).

The Model V was proposed by (Gorenstein et al., 2011) and consists of two factors: Cognitive–Affective and Somatic–Affective. Those dimensions are aligned with Beck’s model for depression, which asserts a triad of dysfunctional cognitions (about oneself, others/world, and the future) that triggers negative affect, behavioral, and somatic responses (see Disner, Beevers, Haigh, & Beck, 2011). For this reason, the BDI-II lists cognitive symptoms like self-criticism (item 8) and self-devaluation (item 14), somatic symptoms like tiredness (item 20) and loss of energy (item 15), and affective symptoms like sadness (item 1) and pessimism (item 2). In the structure of Gorenstein et al. (2011), cognitive and somatic symptoms emerge as two separate factors, namely two specific groups of symptoms. However, the affective symptoms are linked to both factors and do not discriminate a single axis of the symptomatology. Affective symptoms aggregated within cognitive or somatic factors have already been found in different CFA of the BDI and BDI-II (Beck et al., 1996; Brown, Kaplan, & Jason, 2012 Campos & Gonçalves, 2011; Dozois et al., 1998; Finger & Argimon, 2013). Therefore, the current finding is not uncommon because affective symptoms might become cognitive and/or somatic dimensions according to the background and/or participants’ profile (Beck et al., 1996; Steer et al., 1999; Steer, Ball, Ranieri, & Beck, 1997).

In the current research, we noticed that a modified bifactor model was more appropriate for identifying depressive symptoms with the BDI-II in our sample. After scrutinizing possible changes to improve the bifactor Model V and to make it parsimonious, we concluded that the removal of non-significant parameters related to four items of the SA factor did not change the basis of the proposal of Gorenstein et al. (2011). These items now integrate only the general factor in Model V (modified), without belonging to their specific factor as originally designated. Thus, these five items (the four excluded from the specific factor, plus item 20) are restricted to the sum of the total score, contributing exclusively to estimation of the general pattern of depressive symptoms. This way of interpreting the BDI-II factorial structure is not uncommon and has been assumed in other psychometric studies, which have found excellent results (Brouwer et al., 2013; McElroy et al., 2018; Quilty, Zhang, & Bagby, 2010; Ward, 2006).

However, although the presence of the G-factor ensures that the interpretation of the total BDI-II score is valid after the specific factors are partialized, some peculiarities in these specific factors require caution in interpreting the findings. The first is that factor SA had 4 somatic items saturating exclusively in the G-factor (items 15, 16, 18 and 21), in addition to item 20, which was the parameter of analysis *S.I-1*. Hence, the SA dimension had its content altered, since only four items (11, 13, 17 and 19) remained in their original factor, and only one item was explicitly somatic (11, Agitation). For the other three items, which are related to the affective dimension (13, indecision; 17, irritability; 19, difficulties in concentration), there seems to be some question about their adequacy to the factor predicted in the model. It is important to mention that depending on the theoretical emphasis, these items can be classified as cognitive, affective or even a combination of these dimensions (e.g. see distribution of items by factor/model in our Method section, Huang & Chen, 2014 or Wang & Gorenstein, 2013a for more examples). Therefore, even though they were considered Somatic-Affective symptoms by Gorenstein et al. – and also in the original model by Beck et al. (1996) – these items may be understood as behavioral manifestations of cognitive elements (impairment of thinking ability) or as affective symptoms (if the emotional repercussion of the item is emphasized) (e.g. Beck et al., 1996; Huang & Chen, 2014; Steer et al., 1997; Steer et al., 1999; Wang & Gorenstein, 2013a; Ward, 2006). Based on these questions, we found that the SA factor was almost restricted to the interpretation of one type of symptomatology (i.e. the affective), partially changing the characterization of the grouping of somatic and affective symptoms, as conjectured by Gorenstein et al. (2011)

Given the possible theoretical problems of specific factor scores, and especially considering that the bifactor structure proved to be the most appropriate factor solution, our findings show that it is more appropriate to use the BDI-II to identify the total symptom score. This is in accordance with the original instructions of Beck et al. (1996), who recommended the use of total score as the main application of BDI. On the other hand, the findings also suggest that the calculation of specific factor scores is not recommended without first taking into account individuals’ overall factor scores. If it is deemed necessary to use the CA and SA factor scores for some reason (for example, to monitor the effect of clinical interventions), this should be done together with the total score and should respect some caveats – especially regarding the interpretation of the SA score, since the remaining items do not fully reflect the conceptual dimension of the factor. Similar recommendations were made in different studies (e.g. Brouwer et al., 2013; McElroy et al., 2018; Osman, Barrios, Gutierrez, Williams, & Bailey, 2008; Quilty et al., 2010; Subica et al., 2014; Ward, 2006).

We also note that some items had low factor loadings (≤.30). This indicates a weak relationship between the item and its respective specific factor in the presence of the general factor, which is expected (Brouwer et al., 2013). This occurred with items 10 (crying) and 12 (loss of interest) in factor CA, and item 19 (difficulties in concentration) in factor SA. As this study is confirmatory, we chose not to change the composition of the factors, for example, excluding the mentioned items. We made this decision based on the fact that the fit of model V (modified) was satisfactory in different indicators of the CFA. Moreover, any factor change or exclusion of an item would lead to an exploratory procedure of the model of Gorenstein et al. (2011); which was not the objective of the current investigation. Besides this, even with low factor loadings, the parameters of these items remained statistically significant. That is, they contributed to explain the specific variance of the factor and therefore could not be discarded. Another reason is that since the bifactor structure represented the most appropriate solution, the presence of a powerful G factor is common and tends to aggregate most of the variance of the items. Thus, the factor loadings of specific factors are reduced in the presence of the G-factor, even at values well below the expected satisfactory saturation, but this does not determine the incompatibility of the item with its specific factor of origin in the bifactor analyses. e.g. (Brouwer et al., 2013; García-Batista et al., 2018; Osman et al., 2008; Quilty et al., 2010; Ward, 2006).

Especially in relation to these questions regarding the items and representativeness of specific factors, it is plausible to recommend that future studies focus on the identification of other exploratory models for the BDI-II in Brazil. Preferably, we suggest doing this with data from the general population, with a non-clinical profile, in order to verify the existence of alternative models and to enable comparison of findings related to the BDI-II factorial structure.

Some studies have suggested that MI confirms the suitability of the BDI-II’s scores for comparing depressive symptomatology between men and women, but this is not a consensus in the literature. Some investigations have also found invariance by gender (undergraduate and high school students, Contreras-Valdez, Hernández-Guzmán, & Freyre, 2015; college students, Whisman et al., 2012), but the non-invariance has also been detected in other investigations (college students, Wu, 2010; high school students, Wu & Huang, 2014). Probably, cultural and socio-demographic aspects can influence such discrepancy in relation to the BDI-II pattern of answers or even to the depressive symptoms’ expression in different sample profiles (Beck et al., 1996; Dere et al., 2015; Steer et al., 1997; Steer et al., 1999; Wang & Gorenstein, 2013a). It should be noted that the mentioned investigations were not performed with a sample composed of community-dwelling adults, which means this study is the first with such specificity; as far as we know. Nevertheless, we did not find significant gender differences regarding item structure and factors, as well as latent scores for those groups. Therefore, these findings add more evidence of the suitability of contrasting BDI-II scores of depressive symptoms by gender.

The strengths of this study include the use of a large randomized sample of non-clinical adults in the Brazilian Northeast. Besides that, to the best of our knowledge, this is the first application of CFA and MI to examine the BDI-II in Brazil. Notwithstanding such main strengths, the current investigation has some limitations, closely related to those assets. The main set of limitations is related to the fact that we did not estimate the depressive symptoms regarding age groups (like adolescents or elderly) or clinical conditions (depressed versus non-depressed or presence versus absence of chronic diseases). Consequently, as literature reviews showed that the BDI-II model can vary in those samples (Wang & Gorenstein, 2013a; Wang & Gorenstein, 2013b), further research is needed to examine the item structure (CFA and MI) to provide additional evidence of the capacity of the BDI-II assess depressive symptomatology in other population groups.

Even with the set of recommendations on the interpretability of the current findings, our study advances prior research on the factor structure of the BDI-II especially because we estimated the G factor by specifying *S.I-1* models, which ensured that a bifactor model was actually performed (see Eid et al., 2017). These findings provide new insights to analyze the consistency of previous results and show better suitability of the bifactor structure for the BDI-II compared to conventional procedures of bifactor analysis. It is also worth noting that although the interpretation of the total score is based on the relevance of the bifactor structure, it cannot be stated that the BDI-II has only a unidimensional structure. Our results showed that the bifactor models – which are multidimensional – were superior to the unidimensional model (Model I), which has been consistently found in similar research with the BDI-II (Brouwer et al., 2013; Heinrich et al., 2018; McElroy et al., 2018; Subica et al., 2014).

Despite the consistency of the results presented here, our approach did not focus on different possibilities of studying the BDI-II’s psychometric properties. For instance, it would be possible to estimate parameters by using Rasch models to analyze the quality of each item (e.g. Sauer, Ziegler, & Schmitt, 2013). Another option would be to analyze item-to-item association in a network analysis to scrutinize whether symptoms have an autonomous influence on each other (e.g. Bringmann, Lemmens, Huibers, Borsboom, & Tuerlinckx, 2015). Indeed, the presence of several plausible factor structures suggests that distinction between cognitive, affective, and somatic latent factors as causal instance for the BDI-II items may be questionable, since symptoms experienced by patients can be affected by other psychological instances beyond a supposed latent variable (in the current case, depression as a syndrome represented by latent factors). Accordingly, we think that further research should address this issue by questioning the adequacy of the assumption underlying the common latent factor structure approach. Also, it seems relevant to extend the study of the symptoms listed in the BDI-II by using alternative approaches, such as those proposed in the models of item response theory and network analysis, which have already been applied in previous studies (Sauer et al., 2013; Bringmann et al., 2015).

Finally, our results suggest that the BDI-II is a useful instrument for measuring depressive symptoms in community-dwelling people and comparisons by gender in the general population are suitable. Its features of being self-administered and brief are desired to surveys for screening depressive disorders in primary health care settings or even academic studies. Through CFA, we found that it is valid to use the total score for mapping the presence of possible cases of depression. However, we also found that scores of the specific factors should not be used as the first choice. The analysis of severity, based on a total score, seems to be the most appropriate option. Thus, we believe these results may contribute to increasing the chances of early identification of a depressive disorder and the proper referral for health care in Brazil.
